# Supplementary material for: Patterns of Intron Gain and Loss in Fungi
Source: PLoS Biol. 2004 Nov 30;2(12):e422. doi: 10.1371/journal.pbio.0020422 (PMC532390; doi:10.1371/journal.pbio.0020422)
Supplement: Table S1 — Also available at http://genes.mit.edu/NielsenEtAl/. (4.3 MB ZIP). [file pbio.0020422.st001.zip › NielsenEtAl/html/1397.html]

AN5960.1.NCU07830.1.MG05238.1.FG02541.1


```
 CLUSTAL W (1.82) Multiple Sequence Alignments - Introns Inserted


Sequence 1: NCU07830.1	150 aa
Sequence 2: MG05238.1	150 aa
Sequence 3: FG02541.1	151 aa
Sequence 4: AN5960.1	149 aa
Alignment Length: 151 aa
Number Identitical Residues: 130 aa
Alignment Score (without introns) 5009


MG05238.1 	MPPKK-TQRPAQENISLGPQVREG1ELVFGV~ARIFASFNDTFVHVTDLS2GRETICRVT
NCU07830.1	MPPKK-AARPAQENISLGPQIREG~ELVFGV1ARIFASFNDTFVHVTDLS2GRETICRVT
FG02541.1 	MPPKKTATRAPQENISLGPSVRDG1ELVFGV~ARIFASFNDTFVHVTDLS2GRETITRVT
AN5960.1  	MAPKT--KAPAKDNVTLGPLAGDG1KLVFGV~ARIFASFNDTFVHVTDLS2GRETICRVT
          	*.**.    ..::*::***   :* :***** ****************** ***** ***

MG05238.1 	GGMKVKADRDESSPYAAMLAAQDVAARCKELGITALHIKIRATGG~NGTKTPGPGAQSAL
NCU07830.1	GGMKVKADRDESSPYAAMLAAQDVAARCKELGITALHIKIRATGG1NGTKTPGPGAQSAL
FG02541.1 	GGMKVKADRDESSPYAAMLAAQDVAARCKELGINALHIKIRATGG1NGTKTPGPGAQSAL
AN5960.1  	GGMKVKADRDESSPYAAMLAAQDVAARCKELGINALHIKIRATGG1NGTKTPGPGAQSAL
          	*********************************.*********** **************

MG05238.1 	RALARSGMKIGRIEDVTPTPSDSTRRKGGRRGRRL
NCU07830.1	RALARSGMKIGRIEDVTPTPSDSTRRKGGRRGRRL
FG02541.1 	RALARAGMKIGRIEDVTPTPSDSTRRKGGRRGRRL
AN5960.1  	RALARSGMRIGRIEDVTPTPSDSTRRKGGRRGRRL
          	*****:**:**************************
```
